# Combining high-flow nasal cannula oxygen therapy with repeated toilet bronchoscopies for respiratory failure due to excessive infected airway secretions: a case report and series from a non-intensive hospital ward

**DOI:** 10.3389/fmed.2024.1361372

**Published:** 2024-09-25

**Authors:** Filippo Luca Fimognari, Francesco Baffa Bellucci, Flavio Fedele, Simone Scarlata, Giuseppe Armentaro, Angela Sciacqua

**Affiliations:** ^1^Unit of Geriatrics, Department of Medicine, Azienda Ospedaliera Annunziata-Mariano Santo-S. Barbara, Cosenza, Italy; ^2^Unit of Bronchology, Azienda Ospedaliera Annunziata-Mariano Santo-S. Barbara, Cosenza, Italy; ^3^Unit of Internal Medicine, Fondazione Policlinico Universitario Campus Biomedico, Rome, Italy; ^4^Unit of Geriatrics, Azienda Ospedaliero-Universitaria Renato Dulbecco, Catanzaro, Italy; ^5^Department of Medical and Surgical Sciences, University Magna Graecia, Catanzaro, Italy

**Keywords:** fiberoptic bronchoscopy, toilet fiberoptic bronchoscopy, acute respiratory failure, high-flow nasal cannula oxygen therapy, geriatric hospital ward

## Abstract

Fiberoptic bronchoscopy (FBO) has diagnostic or therapeutic purposes but can cause respiratory deterioration, particularly in patients with pre-existing acute respiratory failure (ARF). Non-invasive ventilation (NIV) and high-flow nasal cannula oxygen therapy (HFNC) are used as respiratory support for ARF as well as to prevent significant oxygen deterioration during FBO. The combined use of NIV and early therapeutic FBO to clear retained abundant infected secretions from the airways may be an alternative to intubation and invasive mechanical ventilation (IMV), but no data exist on the combined use of FBO and HFNC. A 78-year-old male patient with ARF secondary to chronic obstructive pulmonary disease (COPD) exacerbation and pneumonia was admitted to our non-intensive geriatric ward. After an initial improvement, his respiratory conditions worsened. While continuing HFNC, he underwent a series of eight FBOs over 9 days, each performed in response to significant decreases in peripheral oxygen saturation (SpO_2_). The goal was to remove copious and occlusive infected secretions from the airways, with each procedure resulting in good SpO_2_ recovery. After etiological targeted antibiotic therapy based on bronchial aspirate, the patient improved and was discharged. Next, six consecutive similar ARF patients were treated using the same strategy of combining HFNC with repeated toilet FBO performed within the ward to clear secretions. All patients showed improvement and were discharged. The combination of HFNC and repeated toilet FBO could be a safe and effective intervention in non-intensive wards to prevent intubation and IMV in frail and elderly patients with ARF secondary to copious and occlusive infected secretions in the airways.

## 1 Introduction

Acute respiratory failure (ARF) is a complex syndrome diagnosed on the basis of a low level of partial pressure of oxygen (pO_2_) in the arterial blood, often resulting from acute illnesses or exacerbations of chronic cardio-pulmonary disorders (1). ARF is a very frequent reason for hospital admission in older patients. In an Italian national survey including patients admitted to hospital after evaluation in the emergency department (ED), it was documented that ARF was the most frequent discharge diagnosis from hospital wards for persons aged ≥75 years (2). In a recent prospective study, ARF predicted short-term death of older patients hospitalized in medical wards after accounting for the severity of critical illness, frailty, delirium, and other confounders (3). Underlying causes that may trigger ARF in elderly patients include congestive heart failure, pneumonia, exacerbation of chronic obstructive pulmonary disease (COPD), sepsis, pulmonary embolism, acute bronchitis, and other conditions. These causes frequently occur in combination due to the patients’ clinical vulnerability (1).

In addition to the prompt identification and treatment of causative illnesses, ARF management requests respiratory support to correct hypoxia and improve respiratory dynamics, such as conventional oxygen therapy (COT), non-invasive mechanical ventilation (NIV), or high-flow nasal cannula oxygen therapy (HFNC). These non-invasive respiratory treatments aim at avoiding escalation to endotracheal intubation and invasive mechanical ventilation (IMV) in the intensive care units (ICU) (4).

High-flow nasal cannula oxygen therapy (HFNC) is strongly indicated for the treatment of type 1 ARF, i.e., hypoxia without hypercapnia (5). A very high flow of heated and humidified gas mixture (oxygen and room air) is administered through soft nasal prongs. The high flow generates a continuous positive pressure in the airways (approximately 5 cmH_2_O at a flow of 60 L/min with a closed patient’s mouth), and a flow-dependent washout of expired gas in the dead space reduces carbon dioxide (CO_2_) (4). Furthermore, due to ease of use and high tolerability, HFNC is particularly suitable for older patients hospitalized in non-intensive settings (4). In addition, it is more effective than COT in preventing adverse events (desaturation, worsening of ARF, and hemodynamic instability) that may sometimes complicate flexible fiberoptic bronchoscopy (FBO) performed for diagnostic or therapeutic purposes (6, 7).

In case of failure of non-invasive respiratory supports, the clinical decision to subject frail elderly patients to IMV may be controversial due to difficulties in predicting prognosis, limited availability of beds in ICUs, and concerns regarding intubation-related complications (1). Thus, combined non-invasive therapeutic strategies should be developed within non-intensive hospital settings to prevent IMV in severe older ARF patients without compromising clinical outcomes (8). For instance, NIV associated with early therapeutic FBO for removing copious occlusive secretions in the airways was found to be a valid alternative to IMV (9).

In this case report and subsequent case series, we describe a strategy combining HFNC with repeated therapeutic FBO, which successfully prevented intubation in ARF secondary to copious infected secretions in the airways.

## 2 Case report and case series

A 78-year-old man presented to the ED because of acute-onset (less than 1 day) worsening of dyspnea and severe ARF on 14 September 2022. The patient’s chronic diseases included type 2 diabetes mellitus (metformin 500 mg *bis in die*), chronic atrial fibrillation (rivaroxaban 20 mg per day and digoxin 0.125 mg per day), COPD (inhaled fluticasone furoate/vilanterol 92/22 μg plus inhaled umeclidinium bromide 55 μg, both once per day), and hypertension (amlodipine 5 mg and enalapril 10 mg). His baseline functional status, before the acute worsening, was normal. On admission to our ward, the patient was afebrile and tachypneic (approximately 22 breaths per minute). Peripheral oxygen saturation (SpO_2_) was 82% with a fraction of inspired oxygen (FiO_2_) of 50%, and arterial blood analysis (ABG) showed type 1 ARF. His heart rate was 135 beats per minute and his blood pressure was 140/70 mmHg. His mental status was normal, vesicular breath sounds were diminished, and a right-sided hemiparesis, mostly involving the upper extremity, was detected. N-terminal pro-brain natriuretic peptide (NT-proBNP) and troponin measurements showed values of 11.296 pg./mL and 5.6 ng/mL, respectively.

Based on chest X-rays (bilateral hilar interstitial and alveolar edema) and head computed tomography (CT, pre-Rolandic left ischemic stroke) previously performed in the ED, an initial diagnosis of ARF secondary to acute congestive heart failure, COPD exacerbation, and possible pneumonia, associated with a left ischemic stroke, was made. NIV support was continued (face mask, bi-level pressure support with expiratory pressure of 6 cmH_2_O, inspiratory pressure of 16 cmH_2_O, and 10 L/min of oxygen flow), but the patient’s respiratory conditions worsened, and he was transferred to the ICU after only 5 h of stay in our ward. In the ICU, NIV was replaced by HFNC (60 L/min, FiO_2_ 60%, and temperature 34°C), and empiric intravenous (IV) piperacillin/tazobactam 4.5 g three times daily was continued. Following overall and respiratory improvement, he was shifted back to our ward 2 days later. Here, clinical improvement continued, and HFNC was replaced by low-flow COT on day 5 of hospital admission. In the subsequent days, he suffered repeated episodes of rectorrhagia with post-hemorrhagic anemia. A contrast-enhanced total-body CT performed on day 11 showed pneumonia (consolidation) in the left lower lobe and, to a lesser extent, in the lateral and posterior segments of the right lower lobe. Piperacillin/tazobactam was stopped, and an empiric antibiotic therapy of IV meropenem 1 g three times per day plus tigecycline 50 mg (after loading dose of 100 mg) twice per day was started. The same CT also highlighted sigmoid wall thickening, suggestive of cancer.

On day 12, his respiratory status seriously worsened (tachypnea and decreased SpO_2_/FiO_2_ ratio), and HFNC (FiO_2_ 60%, 50 L/min) was reintroduced. The day after (day 13), because of further worsened respiratory status, a CT was repeated, which displayed worsening multifocal pneumonia, with increased involvement of the right lower lobe, extension to the middle lobe, and mucoid obstruction of the right lower lobe bronchus. On the same day, he underwent toilet FBO (performed in the bronchoscopy laboratory while continuing HFNC) that removed abundant retained secretions from the airways of both lungs. After this first FBO, the SpO_2_/FiO_2_ ratio immediately increased (Table 1). Rapid polymerase chain reaction testing of the bronchial samples detected *Acinetobacter baumannii* positive for extended-spectrum beta-lactamase (ESBL) and *Klebsiella pneumoniae* carbapenemase (KPC) producing, with results later confirmed by culture. Meropenem and tigecycline were interrupted. Based on antibiotic sensitivity tests, a treatment of IV ceftazidime/avibactam 2.5 g three times per day, IV colistin 4.5 million International Units (IU) twice per day (after 9 million loading dose), and aerosolized colistin (1 million IU with saline three times per day) was started.

**Table 1:** Sequence of FBO and oxygenation values before and after each procedure.

	Before FBO			After FBO			
Day of FBO	SpO_2_	FiO_2_	SpO_2_ /FiO_2_	SpO_2_	FiO_2_	SpO_2_ /FiO_2_	Notes
13	92	88	1.04	98	74	1.32	
14	88	80	1.1	95	50	1.9	
15	92	91	1.01	99	75	1.32	
17	88	75	1.17	97	70	1.38	
18	78	80	0.97	96	80	1.20	
19	84	85	0.98	96	80	1.17	Removal of mucus plug in the right inferior lobar bronchus.
19	83	80	1.03	98	67	1.46	Removal of mucus plug in the right inferior lobar bronchus.
22	93	60	1.55	98	60	1.63	

FBO, fiberoptic bronchoscopy; SpO_2_, peripheral oxygen saturation by pulse oximetry; FiO_2,_ fraction of inspired oxygen. Day of FBO is the day during hospital stay on which FBO was performed (the day of admission to the ward from the emergency department is day 0).

In the following days, however, frequent measurements of the SpO_2_/FiO_2_ ratio identified further episodes of respiratory deterioration due to the accumulation of infected secretions in the airways of both lungs, mainly in the right inferior lobar bronchus. In each of such respiratory deteriorations, FBO was immediately performed during HFNC and always obtained significant recovery of acceptable arterial oxygenation parameters by clearing secretions in the airways (Table 1). On two occasions, a plug in the right inferior lobar bronchus was removed by FBO (Table 1). Overall, he was submitted to eight FBOs during 9 days, and, except for the first, FBO was always performed at the patient’s bedside. Afterward, the patient’s clinical, laboratory, and respiratory conditions improved; he was switched to COT and discharged after 20 days of targeted etiological antibiotic therapy and a 35-day hospital stay. He subsequently underwent surgery for colon cancer.

After this clinical experience, the non-invasive strategy combining HFNC treatment with repeated FBO—performed in response to episodes of oxygen desaturation with the aim of removing abundant infected occlusive secretions from the airways—was applied in our ward to the other six consecutive patients already in treatment with HFNC for severe ARF (Table 2). In all cases, FBO was performed to treat episodes of oxygen desaturation that were refractory to increases in flow and FiO_2_ up to 60 L/min and 80%, respectively. FBO was undertaken within the ward at the patient’s bedside by experienced pulmonologists inserting the flexible bronchoscope through one nostril after removing the ipsilateral soft nasal HFNC prong. During the procedure, HFNC was continued through the other nostril, and flow and FiO_2_ were adjusted in order to maintain a SpO_2_ value of ≥93%. Bronchial aspirates were sent to the microbiology laboratory for etiological determinations. Complications related to the procedure were never observed, and all patients were discharged alive after clinical improvement (Table 2). The patients, or their proxies for those with severe cognitive impairment, signed an informed consent to undergo FBO.

**Table 2:** Case series of patients submitted to HFNC for severe respiratory failure combined with repeated therapeutic (removal of infected secretions) and diagnostic (bronchial aspirate) FBO.

Age (years)	Sex	Diagnosis	Number of FBO (days of FBO)	Outcome	Isolated pathogen(s) (bronchial aspirate)	Comorbidities	Mean SpO_2_ before FBO*	Mean SpO_2_ after FBO*
79	M	Aspiration pneumonia	9 (4, 5, 7, 8, 11, 13, 15, 16, and 17)	Hospice	Respiratory syncytial virus, *Candida albicans*, and *Candida glabrata*.	HHF, AKI, and vascular dementia.	89	94.3
87	M	AB	3 (3, 4, and 6)	Nursing home	*Candida glabrata*.	PD with dementia and obesity.	88.6	93.3
96	F	AB	2 (3, 4)	Home	Nothing.	AF, BRS, and carotid atherosclerotic disease.	92	98
65	F	Exacerbated COPD	2 (1, 2)	Rehabilitation facility	*Acinetobacter baumannii*.	HHF and BRS secondary to previous stroke.	84	96.5
84	M	AB	3 (6, 21, 23)	Rehabilitation facility	*Candida glabrata*, Influenza A, and *Aspergillus*.	AF, decompensated valvular heart disease, BRS, and type 2 DM.	86.3	93.4
74	M	Pneumonia	2 (8, 12)	Hospice	*Pseudomonas Aeruginosa*.	Metastatic lung cancer, PE, and BRS.	88.5	95

HFNC, high-flow nasal cannula oxygen therapy; FBO, fiberoptic bronchoscopy; SpO_2_, peripheral oxygen saturation by pulse oximetry; M, male; F, female; HHF, hypertensive heart failure; AKI, acute kidney injury; AB, acute bronchitis; PD, Parkinson’s disease; AF, atrial fibrillation; BRS, bed rest syndrome; COPD, chronic obstructive pulmonary disease; DM, diabetes mellitus, PE, pulmonary embolism. Days of FBO are the days of hospital stay on which FBO was performed (the day of admission to the ward from the emergency department is day 0). *Means of values recorded before and after each FBO.

## 3 Discussion

FBO has diagnostic and therapeutic purposes, such as removing abundant and infected secretions occluding the airways (8). On the other hand, since FBO can cause acute dynamic narrowing of the airways with worsening hypoxia and secondary cardiovascular distress, concerns exist regarding its use and safety in patients with severe ARF, in whom many clinicians would not undertake FBO without intubation and IMV (10). However, in our patient with severe ARF, recurrent episodes of desaturation were successfully managed without intubation, removing infected secretions by means of repeated FBO. This strategy also allowed the microbiological identification in the cleared secretions of bacteria responsible for the multifocal pneumonia and COPD exacerbation and the subsequent choice of a targeted etiological antibiotic therapy. Even though a community-onset exacerbated COPD was one of the reasons for initial hospital admission, the following hospital stay was presumably complicated by the development of hospital-acquired pneumonia and severe bronchial infection caused by antibiotic-resistant bacteria (*Acinetobacter Baumannii* and *Klebsiella Pneumoniae*).

COT administered through nasal cannula is used during FBO to counteract the risk of respiratory complications related to the procedure, but it may be insufficient to prevent the worsening of hypoxia and hypercapnia in older patients with comorbidities and severe ARF (11). Because this category of individuals is growing worldwide due to population aging, an increasing number of patients is expected to be excluded from FBO and conclusive diagnosis, owing to safety concerns, particularly in those with pO_2_/FiO_2_ below 100 (12). In addition, such critical patients are often hospitalized in non-intensive wards, and FBO could cause further respiratory compromise that could require unintended intubation and ICU transfer (13).

NIV performed during FBO has been extensively studied as a method capable of preventing such FBO-related respiratory derangement (14). HFNC has also been demonstrated to maintain adequate oxygenation levels better than COT (6, 7) and to avoid the loss of end-expiratory lung volume (6) during diagnostic FBO. NIV seems to be superior to HFNC in preventing oxygenation deterioration during FBO in patients with moderate-to-severe ARF (15, 16), particularly when heart failure contributes to ARF and the application of a positive airway pressure is necessary to determine an adequate positive intrathoracic pressure that cannot be assured by HFNC (10). It is unknown, however, whether the benefit-to-risk ratio of FBO, “assisted” by NIV or HFNC, in patients with ARF may increase when an oxygenation improvement is expected as a therapeutic result of the procedure *per se*, for instance when there is the need to remove mucus plugs and infected secretions from the airways (8). Furthermore, comparative studies of NIV and HFNC are lacking in this specific subgroup of patients who require a “therapeutic” FBO for unclogging the airways.

Indeed, a therapeutic combination of NIV and therapeutic FBO may be effective in COPD patients with abundant obstructive secretions (9, 17). In a case–control study, Scala et al. showed that a strategy based on the combination of early FBO for clearing secretions from the airways and NIV was comparable to a strategy based on FBO after IMV in terms of oxygenation values, hospital mortality, duration of hospitalization, and ventilation (9). In a prospective cohort study, NIV combined with a non-invasive strategy to aspirate and clear secretions from the trachea in the first 2 h was at least as effective as IMV and was also associated with lower hospital mortality (17). These combined strategies aim at avoiding intubation and IMV but can be applied only in expert units, not being within the reach of non-intensive medical hospital settings (8). With this regard, our clinical experience seems to be the first ever reported on the combined use of HFNC and repeated toilet FBO in patients with severe ARF sustained by abundant retained bronchial secretions in a non-intensive setting (8). FBO is easier to perform and better tolerated if “assisted” by HFNC compared to NIV, particularly in frail and uncooperative patients hospitalized in non-intensive medical wards (8, 18). HFNC assures several beneficial physiological effects: flow-dependent washout of expired gas in the dead space (reducing CO_2_), generation of a flow-dependent continuous positive pressure in the airways with alveolar recruitment, improved mucociliary clearance by the heated and humidified gas, adequate matching between ventilation and the patient’s inspiratory effort, reduction of airway resistance that could balance the increase that is possibly caused by FBO (4, 10), and augmented end-respiratory lung volume reflecting alveolar recruitment (6). In addition, the fact that, unlike NIV, HFNC can be provided continuously without interruptions—before, during, and after the procedure—could theoretically emphasize the synergy with FBO (4, 8).

In our patients, the removal of secretions from the airways—together with the above-mentioned physiological effects of HFNC—may have accounted for the increased oxygenation obtained after each toilet FBO performed for episodes of significant oxygen desaturation. The peculiarities of these observations are the high number (up to 9) of FBO, the fact that such procedures were performed within the non-intensive ward, and that no procedure-related complications were reported. Furthermore, although our elderly patients were frail and burdened with significant comorbidities, intubation and ICU transfer were avoided, and they all were discharged alive. Another not negligible aspect is the diagnostic value of repeated FBO, which provided occasions to identify in the bronchial aspirate the pathogen(s) responsible for the acute respiratory infection.

This study has limitations. Since this is a real-life clinical observation, no firm conclusion can be drawn, and the combination of HFNC/repeated toilet FBO needs to be tested in a randomized controlled trial to verify whether it may really be superior to other strategies, mostly intubation and IMV, in improving outcomes of frail patients with severe ARF due to abundant retained infected secretions in the airways. We did not measure cough peak flow and did not use a mechanically assisted cough device that would have reduced the need for endoscopic toilets. In our busy hospital setting, arterial oxygenation trajectories were carefully and non-invasively tracked by SpO_2_ /FiO_2_ rather than obtaining a comparable number of partial pressure of oxygen (PaO_2_) measurements by arterial blood analysis. In addition, ROX or modified ROX indexes (19), both incorporating high respiratory rate and poor arterial oxygenation values (SpO_2_ /FiO_2_ or PaO_2_ /FiO_2_, respectively) for the prediction of HFNC failure, were not routinely used to capture the need for therapeutic FBO as an alternative to intubation. It should be mentioned, however, that the reported post-FBO improvements in SpO_2_ /FiO_2_ inevitably resulted in an increase of ROX/modified ROX indexes, and poor arterial oxygenation was found to be a better predictor of failure than a high respiratory rate in patients supported with non-invasive oxygenation strategies (20).

In conclusion, the combination of continuous HFNC treatment and repeated therapeutic FBO could be a safe and effective intervention to apply in non-intensive wards for preventing ICU transfer of older and frail patients with ARF secondary to obstructive infected secretions in the airways.

## Data Availability

The raw data supporting the conclusions of this article will be made available by the authors, without undue reservation.
